# Commentary: Effect of Dead Sea Climatotherapy on Psoriasis; A Prospective Cohort Study

**DOI:** 10.3389/fmed.2020.586418

**Published:** 2020-11-20

**Authors:** Marco Harari

**Affiliations:** Dead Sea and Arava Reseach Center, Masada Institute, Jerusalem, Israel

**Keywords:** Dead Sea, climatotherapy, long-term results, psoriasis, UVB

## Introduction

We read with interest the article of Thomas Emmanuel and colleagues recently published (1). We would like to elaborate and highlight some critical points which might alter the authors' conclusions regarding their statement on long term effects of the treatment.

In the light of currently given treatments for Psoriasis, DSC remains a valuable and attractive option for many reasons. Defined as a natural and selective balneo-phototherapy, DSC should benefit from the recommendations of many authors regarding the necessity to maintain Phototherapy “as an integral part of the dermatology treatment armamentarium” (2).

DSC is an effective therapeutic option with almost no side effects, if realized under strict medical supervision, and proposed through a personalized UVB dosage regimen, adapted to the time of the sun exposure sessions (3, 4). Administered in this manner, this modality can be easily compared to artificial Phototherapy, in mean of cumulative UVB dose received, while controlling the potential long-term risks of UV treatment.

## Comments on Methodology

The methodology of this study was meticulous, with an abundance of parameters, but the doses of Ultra-Violet B (UVB) radiation received by the patients are lacking. It is difficult to understand the results of any study dealing with natural or artificial phototherapy without such measurements (5). Moreover, such quantitative evaluation can optimize benefits of Phototherapy while reducing the risks of any excessive exposure (6).

Prospective cohort studies typically necessitate large sample sizes, while overcoming selection bias and confounding variables. Increasing the sample size tends to reduce the sampling error, making the sample statistic less variable. Using a simple equation including the constant C (dependent of the degree of significance), the standard deviation value (s) and the minimal requested difference (d) for the variable, we obtained the value of 55.2376 for the variable PASI as minimal sample size, i.e., 56 patients. Therefore, we suggest that roughly 60 patients should be enrolled to make a sound conclusion in such a study.

n = 1+ 2C (s/d)^2^ = 1+29.76(5.4/4)(5.4/4) = 55.2376. [C = 14.88; s = 5.4 (PASI standard deviation for average PASI; d = 4 (minimal clinically important difference, MCID = 30%, equivalent to 4 points in PASI value)].

When looking at PASI as primary endpoint, the statistical workup of this study can be divided in two distinct and unequal parts. The first one reports on immediate effects of the procedure on 15 patients, from whom only 10, totally cleared, were enrolled in the follow up. The second one describes the evaluation of 6 of them, 3.3 months after [Table 2, in (1)].

Additionally, the choice of a strict definition of relapse after DSC (“reappearance of visible skin symptom”) should be underlined, taking into consideration the less severe ones used in other clinical trials.

## Comments on Results

In view of our large experience, following regularly patients returning to the Dead Sea for treatment, we can confirm the results of the pioneer study carried out in 2006, on 66 patients. Statistical analysis demonstrated a 23.1 week remission and a duration of therapeutic effect of 33.6 weeks (7) [cited as ref. 16 in (1)].

In this unique work, realized by a team of German dermatologists, patients' follow-up and determination of PASI values was possible for the whole cohort, many weeks after the Dead Sea treatment. Since these times, thousands of Psoriasis patients continued to be treated at the Dead Sea, and evaluation of their symptom-free interval still correlate with this published one.

More and more, Quality of Life (QoL) evaluation is being reported in psoriasis studies. A recent important review (8) succeeded to screen 3,646 publications and reported on 100 trials in which topical (33 items), systemic (18 items), and biologic therapy (39 items) were tested, among phototherapy (9 items) and some other interventions. The most commonly used QoL instrument was, in the vast majority of them (83%), the Dermatology Life Quality Index (DLQI), followed by the 36-Item Short Form Survey (SF-36) (31%), the EuroQoL-5D (EQ-5D) (15%), the Psoriasis Disability Index (14%), and the Skindex (5%). We recommend the use of DLQI in order to assess long-term efficacy of Climatotherapy, while developing definitions of minimal clinically important difference (MCID) for each sub-category, as suggested in the above mentioned review.

Many parameters should be included in a comparison between Balneotherapy, Heliotherapy, or Climatotherapy, Phototherapy and systemic treatment like Biologics. The foremost one should be the type of therapy—continuous (systemic) or intermittent, “as needed.” Such a distinction, important not only for the cost-effectiveness calculation, allows a better understanding of what we are comparing.

A modern evaluation of a treatment, particularly for a chronic disease, should include some remarks on its cost-effectiveness ratio. Many studies published on this topic (2) conclude on the importance of phototherapy in the arsenal of psoriasis therapies, even in the era of biologics. Such an approach help drawing a more complete picture of DSC, adding an important parameter—sometimes decisive for the health insurance strategies.

A recent systematic review (9) reports on 775 studies dealing with the costs associated with managing and treating psoriasis and psoriatic arthritis, in 5 European countries: Germany, Spain, France, Italy, and the United Kingdom. The total annual cost per patient ranged from 2,077 to 13,132 US$ for psoriasis and from 10,924 to 17,050 US$ for psoriatic arthritis. The authors pointed out the fact that the introduction of biologics considerably increased direct and total costs.

Costs of Phototherapy differ between countries but are low. A 2010 analysis reported (in 9, ref. 33.) respectively 3,148 and 7,582 US$ for a year of NB-UVB and PUVA. Authors insisted in a recent study on the sparring effect of Phototherapy in the management of psoriasis (10).

In an interesting work done on a total of 108,790 psoriasis patients (11), the authors determined the cost-per-patient-per-year for an average follow-up time of almost 3 years. The average all-cause healthcare costs reached 12,523 US$ while for patients with moderate-to-severe disease, patients with psoriatic arthritis and patients on biologics they reached 21,481, 23,427, and 29,832 US$, respectively.

Cost of Climatotherapy, which is mainly proposed for moderate-to-severe cases, was barely evaluated in the past, and the authors mentioned one of the papers which dealt on it specifically (ref. 15 in 1.). Dead Sea Climatotherapy direct costs can be easily calculated. They comprise flight, transfer, accommodation and medical supervision. For a patient coming from a European country we calculated the cost of a 4-week treatment around 5,800 US$ (5,000 Euros).

Inspired by the work of Lim, Hamzawi and colleagues (9), we summarize the characteristics of Phototherapy, Biologics, and Dead Sea Climatotherapy (Table 1). We believe that such an overview shed some light and the advantages and limitations of each one of these treatment modalities.

**Table 1:** Comparison of phototherapy, Dead Sea climatotherapy and treatment with biologics for psoriasis patients.

	**Phototherapy**	**Dead Sea climatotherapy**	**Biologics**
Therapy type	Intermittent	Once a year	Continuous
Therapy use	Potentially not limited (UVB)	Not limited	Potentially not limited Necessity to switch treatments frequently
Efficacy:			
- Short-term - Long-term	- Effective - 3 to 6 months	- Highly effective (in 4 weeks) - 8 months but still debated	- Highly effective (in 8–12 weeks) - Effective
Safety concerns:			
- Short-term - Long-term	- Good safety records - Known good safety records	- Good safety records - Lacking data	- Good safety records - Still lacking studies
Availability/Convenience	Necessitates equipment and staff Access not always possible Time consuming therapy	Necessitates to travel abroadOnly 8 months a year (Psoriasis)Necessitates strict medical supervision	High
Monitoring	Regular skin exam needed No lab tests needed	Regular skin exam neededNo lab tests needed	Lab testing before Clinical and lab monitoring
Children, pregnant and nursing mothers	Safe (UVB)	Safe	Not know
Annual cost	Lower cost	Lower cost (all inclusive)	Expensive cost
Reimbursement	Easy to obtain	Not always easy to obtainOnly 4 countries in Europe	Relatively easy to obtain Usually granted
Acceptance/Compliance	Moderate in clinic/hospital High at home	Excellent	Good

## Conclusion

Climatotherapy short-term results are widely recognized and published since many years. However, determination of long-term effects remains a difficult task, with only a few previous publications dealing with this matter and only limited data presented in the present study. We agree with the authors regarding the need of future studies including larger sample sizes in order to reveal the real magnitude of these effects.

## Author Contributions

The author confirms being the sole contributor of this work and has approved it for publication.

## Conflict of Interest

The author declares that the research was conducted in the absence of any commercial or financial relationships that could be construed as a potential conflict of interest.
